# Comparison of Vocal Cord Function in Patients Undergoing Thyroid Surgery With and Without Microscopic Assistance

**DOI:** 10.7759/cureus.116924

**Published:** 2026-09-25

**Authors:** Upinder Kaur, Gurshinderpal Singh, Sumit Prinja

**Affiliations:** 1 Department of Otorhinolaryngology, Guru Gobind Singh Medical College and Hospital, Faridkot, IND

**Keywords:** microscopy, postoperative complications, recurrent laryngeal nerve, thyroidectomy, vocal cords

## Abstract

Introduction

Recurrent laryngeal nerve injury is a complication of thyroid surgery that can affect vocal cord function. Microscopic magnification may improve the visualization of critical anatomical structures and facilitate precise dissections. This study aimed to compare the vocal cord function and postoperative outcomes of patients who underwent thyroid surgery with and without microscopic assistance.

Materials and methods

This hospital-based prospective comparative observational study included patients undergoing hemithyroidectomy or total thyroidectomy at the Department of Otorhinolaryngology. Preoperative and postoperative vocal cord function was assessed using laryngoscopy. Surgical and postoperative outcomes were compared between the groups undergoing microscope-assisted and conventional thyroidectomy. Statistical analysis was performed with p < 0.05 considered statistically significant.

Results

A total of 166 patients were included, comprising 76 (45.8%) and 90 (54.2%) patients in the microscope and conventional groups, respectively. Hemithyroidectomy was performed in 106 (63.9%) patients and was more frequent in microscopy (73.7% vs. 55.6%; p = 0.016). Operative time was significantly longer with microscopy for hemithyroidectomy (114.35 ± 6.7 vs. 96.0 ± 7.59 min; p < 0.001) and total thyroidectomy (130.38 ± 2.7 vs. 110.5 ± 2.64 min; p < 0.001). Transient recurrent laryngeal nerve palsy occurred in three (3.9%) versus 11 (12.2%) patients (p = 0.056), whereas hematoma was significantly lower with microscopy (1.3% vs. 8.9%; p = 0.032). In hemithyroidectomy, any recurrent laryngeal nerve palsy was significantly lower with microscopy (3.6% vs. 16.0%; OR = 0.19, 95% CI: 0.04-0.96; p = 0.046).

Conclusion

Microscopic thyroidectomy may improve recurrent laryngeal nerve preservation, particularly during hemithyroidectomy, although it requires a longer operative time.

## Introduction

Thyroidectomy is a commonly performed surgical procedure in otorhinolaryngology for managing various benign and malignant thyroid disorders [1]. Although thyroid surgery is generally considered safe, the close anatomical relationship between the thyroid gland and important structures, such as the recurrent laryngeal nerve, external branch of the superior laryngeal nerve, parathyroid glands, trachea, and major cervical vessels, makes a meticulous surgical technique essential [2]. Among these structures, the recurrent laryngeal nerve is particularly important because its injury can result in vocal cord paresis or paralysis, leading to hoarseness, breathy voice, vocal fatigue, aspiration, and airway compromise in severe cases [2,3].

The risk of recurrent laryngeal nerve injury varies according to the extent and type of thyroid surgery, underlying pathology, anatomical variations, and surgical expertise. The nerve is especially vulnerable during dissection near the inferior thyroid artery, Berry’s ligament, and thyroid capsule. Traction, compression, thermal injury, clamping, ligation, ischemia, or transection may result in temporary or permanent nerve dysfunction [4,5]. Therefore, identification and preservation of the recurrent laryngeal nerve throughout its course are important components of safe thyroidectomy.

Conventional thyroidectomy is performed using direct visualization without magnification [6]. With advances in surgical techniques, microscope-assisted thyroidectomy has been introduced to enhance the visualization of fine anatomical structures. Magnification may facilitate clearer identification and tracing of the recurrent laryngeal nerve, external branch of the superior laryngeal nerve, and parathyroid glands, thereby allowing more precise dissection and potentially reducing nerve-related complications [6,7]. Previous studies have reported lower rates of recurrent laryngeal nerve dysfunction and other postoperative complications using microscopic techniques [7,8].

In contrast, a systematic review and meta-analysis by Sapalidis et al. [6] found that magnification techniques, including surgical loupes and microscopes, did not significantly reduce the risk of transient recurrent laryngeal nerve injury or transient hypocalcemia compared with direct vision during thyroidectomy. However, the authors emphasized that the available evidence was limited by the small number and sample size of the included studies, highlighting the need for further prospective research. Therefore, this study aimed to assess and compare vocal cord function in patients undergoing thyroid surgery with and without microscopic magnification. The objectives were as follows: (1) to study vocal cord function in patients undergoing conventional thyroid surgery; (2) to study vocal cord function in patients undergoing microscopic thyroid surgery; and (3) to compare vocal cord function between patients undergoing thyroid surgery with and without the use of a microscope.

## Materials and methods

Study design and setting

This was a hospital-based prospective comparative observational study conducted in the Department of Otorhinolaryngology, Guru Gobind Singh Medical College and Hospital (GGSMCH), Faridkot, Punjab, India. The study was conducted over a period of 18 months between June 2024 and December 2025 and included patients undergoing thyroid surgery, including hemithyroidectomy and total thyroidectomy, as part of routine clinical care in the department. The study was approved by the Institutional Ethics Committee of the college (GGS/IEC/12 dated May 23, 2024). Written informed consent was obtained from all participants before their inclusion in the study.

Thyroid operations were performed as part of the routine clinical practice of the department according to the treating surgical team's established clinical and surgical judgment. The investigators did not assign patients to receive microscopic or conventional thyroidectomy as an experimental intervention and did not modify the routine management of the patients for research purposes. Rather, patients undergoing the respective procedures during the study period were prospectively observed, and their demographic, clinical, operative, and postoperative outcomes were systematically recorded. Subsequent analysis compared outcomes between patients who underwent thyroidectomy with microscopic magnification and those who underwent conventional thyroidectomy without microscopic magnification.

Study population

The study population comprised adult patients undergoing thyroid surgery in the Department of Otorhinolaryngology. Both hemithyroidectomy and total thyroidectomy were performed. Patients of either sex, aged 18-60 years, who were planned for thyroid surgery and had normal vocal cord function on preoperative laryngoscopic examination were considered eligible for inclusion. Patients were included irrespective of the indication for thyroid surgery, provided they fulfilled the predefined eligibility criteria. Patients who had a history of previous neck surgery, previous irradiation to the lower neck or upper chest, or advanced thyroid malignancy were excluded. Patients with pre-existing vocal cord dysfunction or abnormal vocal cord mobility on preoperative laryngoscopy were also excluded, as the study aimed to assess vocal cord function following index thyroid surgery.

Sample size estimation

An a priori sample size estimation was performed using G*Power software, version 3.1.9.7 (Heinrich-Heine-Universität Düsseldorf, Düsseldorf, Germany), using a two-sample proportion model. The calculation was based on a previously reported cumulative recurrence rate of nerve palsy of 11% among patients undergoing thyroid surgery with and without microscopic magnification [9]. An 80% statistical power, 95% confidence level, corresponding to an alpha level of 0.05, and an allowable margin of error of 5% were considered. The estimated minimum sample size was 151 patients. Allowing for approximately 10% potential loss to follow-up, incomplete records, or missing data, the final target sample size was increased to 166 patients. A total of 166 patients constituted the final study population. Of these, 76 underwent thyroid surgery with microscopic magnification, and 90 underwent conventional thyroid surgery without microscopic magnification.

Preoperative evaluation

All patients underwent routine preoperative assessment according to the established departmental protocol. A detailed clinical history and physical examination were performed, followed by routine otorhinolaryngological examination. Relevant investigations included thyroid function tests, serum calcium estimation, ultrasonography of the neck, and fine-needle aspiration cytology (FNAC) where clinically indicated. A preoperative laryngoscopic examination was performed to document baseline vocal cord mobility and exclude patients with pre-existing vocal cord dysfunction. Demographic and clinical variables were recorded prospectively, including age, sex, residence, socioeconomic status, presenting complaints, duration of symptoms, thyroid functional status, FNAC findings, and relevant comorbidities. These variables were subsequently used to describe the study population and compare the two surgical groups.

Surgical procedure

All thyroidectomies were performed according to the routine surgical procedures followed by the Department of Otorhinolaryngology. The investigators did not introduce a separate experimental or surgical protocol. The surgical approach was based on standard departmental practice, and the use or non-use of microscopic magnification reflected the operative technique used in routine clinical practice. A transverse horizontal incision was made in a natural skin crease below the collar line, extending between the sternocleidomastoid regions. The incision was carried through the skin and subcutaneous tissues, followed by elevation of the subplatysmal flaps. A midline incision was then made in the fascia separating the strap muscles, and the strap muscles were separated to expose the thyroid gland.

During thyroidectomy, particular attention was given to the identification and preservation of the recurrent laryngeal nerve, parathyroid glands, and their vascular supply, and the external branch of the superior laryngeal nerve. The inferior thyroid artery was identified along its anatomical course, and the recurrent laryngeal nerve was actively identified using established anatomical landmarks. Simon’s triangle, bounded by the esophagus medially, carotid artery laterally, and inferior thyroid artery superiorly, was used as an anatomical landmark for recurrent laryngeal nerve identification. Following identification, the nerve was carefully dissected and preserved throughout the thyroidectomy [10].

The parathyroid glands were carefully identified during thyroid dissection, and their vascular supply was preserved wherever possible. The superior and inferior parathyroid glands were identified according to their anatomical relationship to the recurrent laryngeal nerve and thyroid vascular pedicles, with meticulous dissection performed to maintain their blood supply. Preservation of the parathyroid glands and their vascularity was undertaken to minimize the risk of postoperative hypocalcemia and hypoparathyroidism. When a parathyroid gland could not be preserved in situ because of inadvertent removal or loss of vascularity, the gland was subjected to frozen-section examination for confirmation of parathyroid tissue. Following confirmation, the devascularized gland was fragmented and autotransplanted into the forearm musculature, according to the departmental surgical protocol [11]. The external branch of the superior laryngeal nerve was identified by dissection along Joll's triangle, medial to the superior thyroid pedicle. The individual branches of the superior thyroid artery and vein were identified and ligated separately to minimize the possibility of injury to the external branch of the superior laryngeal nerve [12].

Microscopic thyroidectomy

In patients undergoing microscopic thyroidectomy, the operating microscope was introduced after exposure of the thyroid gland and separation of the strap muscles. It was used during the critical stages of thyroid dissection to provide magnified visualization of the operative field and facilitate precise identification and preservation of the recurrent laryngeal nerve, parathyroid glands, and their vascular supply, and the external branch of the superior laryngeal nerve. The use of magnification was particularly helpful during fine dissection around the recurrent laryngeal nerve and parathyroid glands, where small-caliber vascular and neural structures require enhanced visualization. Published studies on microscopic thyroidectomy have reported the use of approximately 4×-6× magnification to facilitate identification and preservation of the fine hilar vessels of the parathyroid glands [13].

The operating microscope used in the present study was a ZEISS operating microscope (Carl Zeiss AG, Oberkochen, Germany), equipped with an objective lens system and adjustable magnification. During microscopic thyroidectomy, we adjusted magnification according to the operative step. Lower magnification was used to visualize the broader operative field during initial dissection, whereas higher magnification was used for delicate dissection around the recurrent laryngeal nerve, inferior thyroid artery branches, and parathyroid glands for better clarity for readers without any confusion. During microscopic dissection, magnification was adjusted according to the operative step. Lower magnification permitted visualization of the broader operative field during initial dissection, whereas higher magnification was used for delicate dissection around the recurrent laryngeal nerve, inferior thyroid artery branches, and parathyroid glands. Therefore, the principal difference between the microscopic and conventional groups was the use of operative magnification during critical stages of thyroid dissection. The incision, exposure, extent of thyroid resection, identification of anatomical landmarks, hemostasis, and wound closure followed the routine departmental thyroidectomy protocol described above. The microscope was used as an adjunct to direct surgical visualization rather than as a separate experimental surgical procedure.

Conventional thyroidectomy

Patients undergoing conventional thyroidectomy were operated on using the same routine departmental thyroidectomy approach without microscopic magnification. The thyroid gland was exposed following the standard cervical approach, and the recurrent laryngeal nerve, parathyroid glands, and external branch of the superior laryngeal nerve were identified and preserved using direct surgical visualization and established anatomical landmarks. Thus, the study did not compare two independently designed experimental operations. Instead, it compared the outcomes observed following two routinely practiced approaches to thyroidectomy: microscopic and conventional thyroidectomy.

Assessment of vocal cord function

Preoperative laryngoscopy was performed to establish normal baseline vocal cord mobility in all the patients. Postoperative vocal cord function was evaluated using laryngoscopy at predefined follow-up intervals. Assessments were performed on postoperative days 1, 7, 30, 90, and 180. Vocal cord mobility was assessed and documented as normal or impaired based on the laryngoscopic findings. Patients who demonstrated postoperative weakness or immobility of one or both vocal cords underwent serial follow-up. Recovery of vocal cord mobility during follow-up was recorded, whereas persistent dysfunction at 6 months was considered a permanent recurrent laryngeal nerve injury according to the study protocol.

Operative and postoperative assessment

Operative details were prospectively documented, including the type of thyroidectomy, operative time, identification of the recurrent laryngeal nerve, and relevant intraoperative observations. Postoperative assessments included vocal cord mobility, occurrence and type of recurrent laryngeal nerve palsy, postoperative complications, and duration of hospital stay. Demographic, clinical, operative, and laryngoscopic findings were recorded using a predesigned data collection proforma. The primary outcome of interest was postoperative vocal cord function. Secondary observations included recurrent laryngeal nerve identification, transient or persistent recurrent laryngeal nerve palsy, operative time, postoperative complications, and hospital stay.

Data collection and management

All study observations were prospectively recorded using a standardized, predesigned proforma. The data included demographic characteristics, clinical findings, preoperative investigations, operative characteristics, and postoperative laryngoscopic findings at each scheduled follow-up assessment. The collected information was subsequently entered into Microsoft Excel (Microsoft Corporation, Redmond, USA) for data organization and then transferred for statistical analysis.

Statistical analysis

Statistical analysis was performed using IBM SPSS Statistics version 25.0 (IBM Corp., Armonk, New York, USA). The statistical analysis plan specified that continuous variables were summarized as mean ± standard deviation and compared between the microscopic and conventional groups using the independent-samples t-test. Cohen's d was calculated to quantify the effect size for continuous variables. Categorical variables were expressed as frequencies and percentages. Comparisons between groups were performed using Pearson's chi-square test. Fisher's exact test was used when the expected cell counts were less than five. Cramer's V was calculated as the effect size for categorical variables. Subgroup analyses of recurrent laryngeal nerve palsy according to surgical type and high-risk categories were expressed using odds ratios with 95% confidence intervals. All statistical tests were two-sided, and a p < 0.05 was considered statistically significant.

## Results

A total of 166 patients were included, of whom 76 (45.8%) underwent thyroid surgery with a microscope and 90 (54.2%) without a microscope. The mean age was significantly lower in the microscope group than in the non-microscope group (31.45 ± 7.32 vs. 34.15 ± 8.65 years; p = 0.032). There was no significant difference in sex distribution between the groups (p = 0.517). However, residence differed significantly, with a higher proportion of urban patients in the microscope group (p = 0.004). All patients presented with neck swelling, while neck pain was more frequent in the microscope group (p = 0.030). Symptom duration also differed significantly between the groups (p < 0.001). Preoperative thyroid status and FNAC diagnosis were comparable between the groups (p = 0.386 and p = 0.693, respectively) (Table 1).

**Table 1:** Clinical and demographic characteristics of the study population. Data are presented as n (%) for categorical variables and mean ± standard deviation (SD) for continuous variables; p-values were derived from the independent-samples t-test for age and Pearson's chi-square test for categorical variables. Fisher-Freeman-Halton exact test was used when expected cell counts were <5. Effect sizes are reported as Cohen's d for continuous variables and Cramer's V for categorical variables. *p < 0.05 was considered statistically significant. FNAC: fine-needle aspiration cytology.

Variable	Category	Total (n = 166)	With microscope (n = 76)	Without microscope (n = 90)	Test value	p-value	Effect size
Age (years)	Mean ± SD	32.45 ± 8.12	31.45 ± 7.32	34.15 ± 8.65	t = -2.16	0.032*	Cohen's d = 0.335
Sex	Male	70 (42.2%)	30 (39.5%)	40 (44.4%)	χ² = 0.42	0.517	V = 0.050
	Female	96 (57.8%)	46 (60.5%)	50 (55.6%)			
Residence	Urban	53 (31.9%)	33 (43.4%)	20 (22.2%)	χ² = 8.50	0.004*	V = 0.226
	Rural	113 (68.1%)	43 (56.6%)	70 (77.8%)			
Symptoms	Neck swelling	166 (100%)	76 (100%)	90 (100%)	N/A	N/A	N/A
	Neck pain	17 (10.2%)	12 (15.8%)	5 (5.6%)	χ² = 4.70	0.030*	V = 0.168
	Dysphagia	17 (10.2%)	10 (13.2%)	7 (7.8%)	χ² = 1.30	0.254	V = 0.089
Symptoms duration	<1 month	56 (33.7%)	20 (26.3%)	36 (40.0%)	χ² = 17.95	< 0.001*	V = 0.233
	1–3 months	86 (51.8%)	52 (68.4%)	34 (37.8%)			
	>3 months	24 (14.5%)	4 (5.3%)	20 (22.2%)			
Thyroid status	Euthyroid	126 (75.9%)	61 (80.3%)	65 (72.2%)	Fisher-Freeman-Halton exact test	0.386	V = 0.076
	Hypothyroid	34 (20.5%)	12 (15.8%)	22 (24.4%)			
	Hyperthyroid	6 (3.6%)	3 (3.9%)	3 (3.3%)			
FNAC diagnosis	Colloid goitre	126 (75.9%)	55 (72.4%)	71 (78.9%)	Fisher-Freeman-Halton exact test	0.693	V = 0.054
	Medullary carcinoma	6 (3.6%)	3 (3.9%)	3 (3.3%)			
	Follicular carcinoma	17 (10.2%)	8 (10.5%)	9 (10.0%)			
	Papillary carcinoma	17 (10.2%)	10 (13.2%)	7 (7.8%)			

Hemithyroidectomy was performed in 106 (63.9%) patients and was significantly more frequent in the microscope group than in the non-microscope group (p = 0.016). Operative time was significantly longer with microscopic surgery for both hemithyroidectomy (114.35 ± 6.70 vs. 96.00 ± 7.59 min; p < 0.001) and total thyroidectomy (130.38 ± 2.70 vs. 110.50 ± 2.64 min; p < 0.001). Transient recurrent laryngeal nerve palsy occurred in three (3.9%) patients with the microscope compared with 11 (12.2%) without it, although the difference was not statistically significant (p = 0.056). Permanent recurrent laryngeal nerve palsy occurred in two (2.6%) and four (4.4%) patients, respectively (p = 0.531). Hematoma was significantly less frequent in the microscope group (p = 0.032), whereas seroma and wound infection did not differ significantly (Table 2).

**Table 2:** Surgical parameters and postoperative complications according to microscope use. Data are presented as n (%) for categorical variables and mean ± SD for operative time. Operative time was compared using the independent-samples t-test. Categorical variables were compared using Pearson's chi-square test. Effect sizes are reported as Cohen's d for continuous variables and Cramer's V for categorical variables. *p < 0.05 was considered statistically significant. RLN: recurrent laryngeal nerve.

Variable	Category	Total (n = 166)	With microscope (n = 76)	Without microscope (n = 90)	Test value	p-value	Effect size
Surgery type	Hemithyroidectomy	106 (63.9%)	56 (73.7%)	50 (55.6%)	χ² = 5.86	0.016*	V = 0.188
	Total thyroidectomy	60 (36.1%)	20 (26.3%)	40 (44.4%)			
Operative time (minutes) (Mean ± SD)	Hemithyroidectomy	105.69 ± 11.62	114.35 ± 6.7	96.0 ± 7.59	t = 13.15	< 0.001*	Cohen's d = 2.56
	Total thyroidectomy	117.13 ± 9.81	130.38 ± 2.7	110.5 ± 2.64	t = 27.08	< 0.001*	Cohen's d = 7.42
RLN palsy	Transient	14 (8.4%)	3 (3.9%)	11 (12.2%)	χ² = 3.65	0.056	V = 0.148
	Permanent	6 (3.6%)	2 (2.6%)	4 (4.4%)	χ² = 0.39	0.531	V = 0.049
Complications	Seroma	12 (7.2%)	4 (5.3%)	8 (8.9%)	χ² = 0.80	0.370	V = 0.070
	Hematoma	9 (5.4%)	1 (1.3%)	8 (8.9%)	χ² = 4.60	0.032*	V = 0.166
	Wound infection	5 (3.0%)	1 (1.3%)	4 (4.4%)	χ² = 1.38	0.240	V = 0.091

Among patients undergoing hemithyroidectomy, recurrent laryngeal nerve palsy occurred in two (3.6%) patients in the microscope group and eight (16.0%) in the non-microscope group, with significantly lower odds of palsy with microscopic surgery (OR = 0.19, 95% CI: 0.04-0.96; p = 0.046). Among patients undergoing total thyroidectomy, recurrent laryngeal nerve palsy occurred in three (15.0%) and seven (17.5%) patients, respectively, with no significant difference (OR = 0.83, 95% CI: 0.19-3.64; p = 0.808) (Table 3).

**Table 3:** Subgroup analysis of recurrent laryngeal nerve palsy according to type of thyroid surgery and microscope use. Data are presented as n (%); odds ratios (ORs) with 95% confidence intervals (CIs) were used to assess the association between microscope use and RLN palsy within surgical subgroups; p-values were derived from Fisher's exact test because expected cell counts were <5. Effect size was assessed using Cramer's V. *p < 0.05 was considered statistically significant. RLN: recurrent laryngeal nerve.

Surgery type	Microscope use	Total N	RLN palsy (Any), n (%)	Transient, n (%)	Permanent, n (%)	OR (95% CI)	p-value	Effect size
Hemithyroidectomy (n=106)	With	56 (52.8%)	2 (3.6%)	2 (3.6%)	0 (0.0%)	0.19 (0.04 - 0.96)	0.046*	V = 0.209
	Without	50 (47.2%)	8 (16.0%)	6 (12.0%)	2 (4.0%)			
Total thyroidectomy (n=60)	With	20 (33.3%)	3 (15.0%)	2 (10.0%)	1 (5.0%)	0.83 (0.19 - 3.64)	0.808	V = 0.031
	Without	40 (66.7%)	7 (17.5%)	4 (10.0%)	3 (7.5%)			

In the high-risk subgroups, recurrent laryngeal nerve palsy occurred in three (15.0%) and seven (17.5%) patients who underwent total thyroidectomy, two (9.5%) and five (26.3%) patients with malignant disease, and two (16.7%) and five (22.7%) hypothyroid patients in the microscope and non-microscope groups, respectively. None of these differences were statistically significant (Table 4).

**Table 4:** Subgroup analysis of recurrent laryngeal nerve palsy in high-risk patient subgroups according to microscope use. Data are presented as n (%); odds ratios (ORs) with 95% confidence intervals (CIs) were calculated for the association between microscope use and RLN palsy within each high-risk subgroup; p-values were derived from Fisher's exact test because expected cell counts were <5. Effect size was assessed using Cramer's V; p < 0.05 was considered statistically significant. RLN: recurrent laryngeal nerve.

High-risk subgroup	Microscope use	Total N	RLN palsy, n (%)	OR (95% CI)	p-value	Effect Size
Total thyroidectomy (N = 60)	With	20 (33.3%)	3 (15.0%)	0.83 (0.19 – 3.64)	0.808	0.031
	Without	40 (66.7%)	7 (17.5%)			
Malignant cases (Medullary + Follicular + Papillary) (n = 40)	With	21 (52.5%)	2 (9.5%)	0.29 (0.05 – 1.72)	0.226	0.221
	Without	19 (47.5%)	5 (26.3%)			
Hypothyroid patients (n = 34)	With	12 (35.3%)	2 (16.7%)	0.68 (0.11 – 4.18)	0.677	0.072
	Without	22 (64.7%)	5 (22.7%)			

The longitudinal analysis demonstrated a progressive decline in impaired vocal cord function following thyroid surgery across all groups. On day 1, impairment was observed in three (15.0%) and seven (17.5%) patients undergoing total thyroidectomy with and without a microscope, respectively, compared with two (3.6%) and eight (16.0%) after hemithyroidectomy. By day 90, impairment decreased to one (5.0%), three (7.5%), zero (0.0%), and two (4.0%), respectively, with similar findings at day 180. Vocal cord impairment progressively improved during follow-up, with the lowest persistent impairment observed after microscopic hemithyroidectomy (Figure 1).

**Figure 1:**
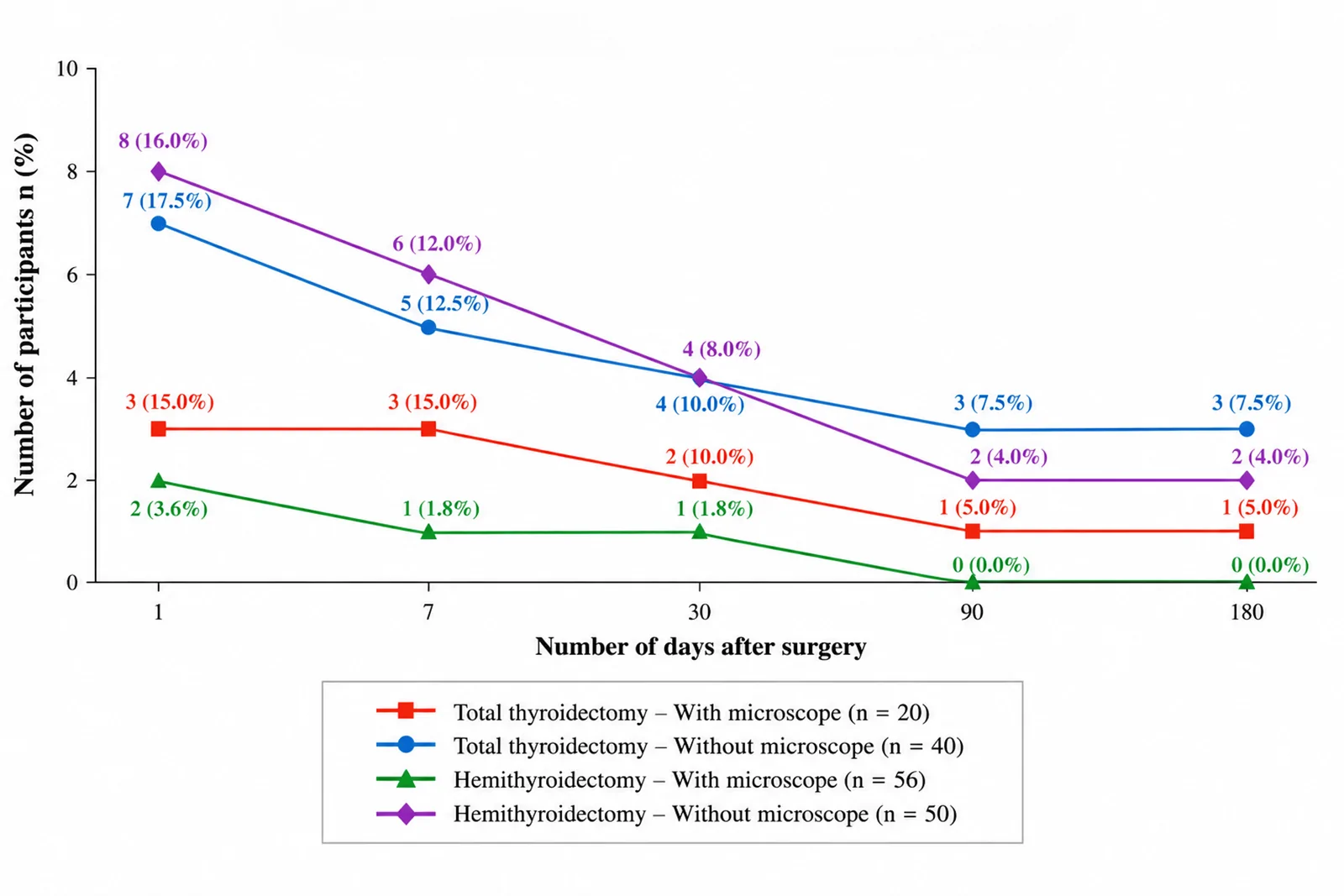
Longitudinal analysis of impaired vocal cord function after thyroid surgery at different time intervals.

## Discussion

The present study evaluated the role of microscopic magnification in thyroid surgery, with particular emphasis on postoperative recurrent laryngeal nerve function. The findings suggest that microscopic magnification may offer an advantage in reducing recurrent laryngeal nerve palsy, particularly during hemithyroidectomy, although this benefit was not consistently demonstrated across all surgical procedures. The findings also indicate that microscopy may improve the precision of dissection but can be associated with increased operative time.

The lower incidence of transient recurrent laryngeal nerve palsy observed with microscopic surgery may be explained by improved visualization of the nerve and its surrounding anatomical structures. Magnification allows the surgeon to distinguish the recurrent laryngeal nerve from adjacent connective tissue and small vascular structures more clearly, potentially reducing inadvertent traction, compression, or injury during thyroid mobilization [7]. This is particularly relevant because recurrent laryngeal nerve injury remains one of the most important complications of thyroid surgery and can result in temporary or permanent vocal cord dysfunction [4,14]. Published microscopic thyroidectomy studies have similarly emphasized the value of magnification for identifying and preserving delicate anatomical structures, including the recurrent laryngeal nerve and parathyroid glands [8,15].

In the present study, the apparent benefit of microscopic magnification was more pronounced among patients who underwent hemithyroidectomy, whereas no significant difference was observed following total thyroidectomy. This finding should be interpreted cautiously because previous studies have included heterogeneous thyroidectomy populations, and evidence directly comparing the effect of magnification according to the extent of thyroid resection remains limited [16]. This difference may be related to variations in the extent and complexity of the surgical dissection. Total thyroidectomy involves bilateral exploration and consequently exposes both the recurrent laryngeal nerves and the parathyroid glands to surgical manipulation [17]. In addition, the underlying pathology and extent of thyroid enlargement or malignancy may influence the difficulty of nerve identification. Therefore, magnification alone may not eliminate the multiple patient- and procedure-related factors that contribute to recurrent laryngeal nerve injury.

These findings are broadly comparable with those of the systematic review and meta-analysis by Sapalidis et al. [6], who evaluated randomized controlled trials comparing magnification techniques with direct vision during thyroid surgery. The authors concluded that magnification techniques were safe but did not demonstrate a statistically significant reduction in transient recurrent laryngeal nerve injury compared to direct visualization. They also emphasized that the available evidence was limited by the relatively small number of patients and studies included. This is consistent with the present findings, in which the overall advantage of microscopy was not uniform, despite a favorable trend in recurrent laryngeal nerve outcomes.

The significant reduction in recurrent laryngeal nerve palsy observed among patients undergoing hemithyroidectomy is clinically relevant. This suggests that microscopic visualization may be particularly beneficial when meticulous dissection around the recurrent laryngeal nerve is required. The absence of a similar effect in total thyroidectomy indicates that the potential benefit should not be generalized to every type of thyroid operation. Rather, microscopy may be most useful as an adjunct in selected procedures or patients in whom precise identification of the nerve is technically challenging.

Microscopic surgery was associated with longer operative duration. This may be explained by the additional time required for microscope positioning, magnification adjustment, and meticulous dissection under magnified visualization. Although prolonged operative time is a practical disadvantage, additional time may be acceptable when it facilitates the safer identification of critical anatomical structures. Previous studies on microscopic thyroidectomy have reported that magnification permits detailed dissection of the recurrent laryngeal nerve and parathyroid structures but requires a more meticulous operative technique [13].

Similarly, Jain et al. [16] emphasized that adaptation to microscopic surgery involves a learning curve and that surgeons who are not accustomed to microscope use may initially require longer operative times. However, evidence is not consistent; Seven et al. [18], in a prospective controlled study comparing microscopic and conventional thyroidectomy, reported no significant difference in operative time between the two techniques. Thus, the increased operative duration observed in the present study may reflect the meticulous nature of microscopic dissection, as well as surgeon experience and familiarity with the technique, rather than an inherent disadvantage of microscopic thyroidectomy.

The lower incidence of postoperative hematoma in the microscopic group may reflect improved visualization of small vessels and more precise hemostasis. However, this finding should be interpreted cautiously because postoperative bleeding is influenced by several factors unrelated to magnification. The lack of a significant difference in most postoperative complications suggests that the potential benefit of microscopic magnification may primarily relate to improved visualization and precise identification of critical anatomical structures rather than a broad reduction in postoperative morbidity. This finding is consistent with the findings of Sapalidis et al. [6], who reported that magnification techniques did not significantly reduce transient recurrent laryngeal nerve injury or transient hypocalcemia compared with direct vision.

Clinical implications

From a clinical perspective, microscopic magnification may be considered a useful adjunct to thyroid surgery, particularly when meticulous identification and preservation of the recurrent laryngeal nerve and parathyroid glands are required. The findings support the potential value of microscopy during hemithyroidectomy, while the absence of consistent benefit across all procedures indicates that microscopy should not be regarded as a replacement for sound anatomical knowledge and careful surgical technique. The additional operative time and requirement for appropriate equipment and surgical familiarity should also be considered when implementing microscopic thyroidectomy.

Limitations

This study was conducted at a single center, which may have limited the generalizability of the findings. Its observational comparative design also allows the possibility of selection and confounding bias because the surgical technique was not randomly assigned. Differences in baseline characteristics and the type of thyroid surgery between groups may have influenced the observed outcomes. The relatively limited number of recurrent laryngeal nerve palsy events also restricts the ability to draw definitive conclusions regarding permanent nerve injury and subgroup effects. In addition, surgeon experience, learning curve, and variation in the use of microscopic magnification could not be fully assessed. Larger multicenter prospective studies with standardized surgical protocols and longer follow-up are therefore required to establish the clinical effectiveness of microscopic magnification in thyroid surgery.

## Conclusions

Microscopic thyroidectomy appears to offer a potential advantage in preserving recurrent laryngeal nerve function, particularly during hemithyroidectomy, through enhanced visualization and precise anatomical dissections. However, this benefit was not consistently demonstrated across all surgical procedures or in the presence of postoperative complications. Microscopic surgery may require a longer operative time and technical expertise. Therefore, microscopy can be considered a useful adjunct in thyroid surgery, especially in anatomically challenging cases. However, larger prospective multicenter studies are warranted to establish its clinical effectiveness.

## References

[1] Sosa JA, Hanna JW, Robinson KA, Lanman RB (2013). Increases in thyroid nodule fine-needle aspirations, operations, and diagnoses of thyroid cancer in the United States. Surgery.

[2] Pradeep PV, Jayashree B, Harshita SS (2012). A closer look at laryngeal nerves during thyroid surgery: a descriptive study of 584 nerves. Anat Res Int.

[3] Erfan S, Saha S, Guha R, Sen I, Kulkarni S (2024). Varying course of external branch of superior laryngeal nerve (EBSLN) and recurrent laryngeal nerve (RLN) in thyroidectomy-an observational study. Indian J Otolaryngol Head Neck Surg.

[4] Uludağ M, Tanal M, İşgör A (2018). A review of methods for the preservation of laryngeal nerves during thyroidectomy. Sisli Etfal Hastan Tip Bul.

[5] Zakaria HM, Al Awad NA, Al Kreedes AS, Al-Mulhim AM, Al-Sharway MA, Hadi MA, Al Sayyah AA (2011). Recurrent laryngeal nerve injury in thyroid surgery. Oman Med J.

[6] Sapalidis K, Papanastasiou A, Fyntanidou V (2019). Comparison between magnification techniques and direct vision in thyroid surgery: a systematic review and meta-analysis. Medicina (Kaunas).

[7] Davidson BJ, Guardiani E, Wang A (2010). Adopting the operating microscope in thyroid surgery: safety, efficiency, and ergonomics. Head Neck.

[8] Palled KP (2024). Microscopic thyroidectomy and its outcomes: retrospective analysis of 25 cases. Karnataka J Surg.

[9] Gautam H, Kumar V, Maurya D (2020). A comparative study of thyroid surgery with and without a microscope. Saudi J Otorhinolaryngol Head Neck Surg.

[10] Varaldo E, Ansaldo GL, Mascherini M, Cafiero F, Minuto MN (2014). Neurological complications in thyroid surgery: a surgical point of view on laryngeal nerves. Front Endocrinol (Lausanne).

[11] Haugen BR, Alexander EK, Bible KC (2016). 2015 American Thyroid Association management guidelines for adult patients with thyroid nodules and differentiated thyroid cancer: the American Thyroid Association Guidelines Task Force on Thyroid Nodules and Differentiated Thyroid Cancer. Thyroid.

[12] Athavale PK, Bokare BD, Ekhar VR, Mahore DM (2013). Identification and preservation of external branch of superior laryngeal nerve in thyroidectomy. Int J Phonosurg Laryngol.

[13] Kumar A, Tyagi AK, Varshney S, Malhotra M, Priya M (2019). Microscopic thyroidectomy: a prudent option. Indian J Otolaryngol Head Neck Surg.

[14] Ojha T, Kansara A, Prasad S, Kansara A (2023). Intra operative recurrent laryngeal nerve monitering during thyroid surgery - is it worth?. Indian J Otolaryngol Head Neck Surg.

[15] Nielsen TR, Andreassen UK, Brown CL, Balle VH, Thomsen J (1998). Microsurgical technique in thyroid surgery--a 10-year experience. J Laryngol Otol.

[16] Jain S, Gupta N, Gupta R, Jain A, Naidu TS (2020). Microscopic thyroidectomy: the way we do it. Indian J Otolaryngol Head Neck Surg.

[17] Ye Y, Wang X, Liu Y (2026). Different approaches of dissecting the recurrent laryngeal nerve during total thyroidectomy affect postoperative parathyroid function. BMC Surg.

[18] Seven H, Calis AB, Vural C, Turgut S (2005). Microscopic thyroidectomy: a prospective controlled trial. Eur Arch Otorhinolaryngol.

